# Analyzing adjuvant radiotherapy suggests a non monotonic radio-sensitivity over tumor volumes

**DOI:** 10.1186/1471-2164-9-S2-S9

**Published:** 2008-09-16

**Authors:** Jack Y Yang, Andrzej Niemierko, Mary Qu Yang, Youping Deng

**Affiliations:** 1 Department of Radiation Oncology, Massachusetts General Hospital and Harvard Medical School, Boston, MA 02114, USA; 2 National Human Genome Research Institute, National Institutes of Health, Bethesda, MD 20852, USA; 3 Department of Biological Sciences, Bioinformatics and Cancer Biology Laboratory, University of Southern Mississippi, Hattiesburg, MS 39406, USA

## Abstract

**Background:**

Adjuvant Radiotherapy (RT) after surgical removal of tumors proved beneficial in long-term tumor control and treatment planning. For many years, it has been well concluded that radio-sensitivities of tumors upon radiotherapy decrease according to the sizes of tumors and RT models based on Poisson statistics have been used extensively to validate clinical data.

**Results:**

We found that Poisson statistics on RT is actually derived from bacterial cells despite of many validations from clinical data. However cancerous cells do have abnormal cellular communications and use chemical messengers to signal both surrounding normal and cancerous cells to develop new blood vessels and to invade, to metastasis and to overcome intercellular spatial confinements in general. We therefore investigated the cell killing effects on adjuvant RT and found that radio-sensitivity is actually not a monotonic function of volume as it was believed before. We present detailed analysis and explanation to justify above statement. Based on EUD, we present an equivalent radio-sensitivity model.

**Conclusion:**

We conclude that radio sensitivity is a sophisticated function over tumor volumes, since tumor responses upon radio therapy also depend on cellular communications.

## Background

Radiotherapy (RT) and surgery are proven methods of treating cancer patients. RT plays an important roles in long-term control of tumors and has been combined with surgery or chemotherapy in addition to its role as a stand-alone therapy.

Tumor responses to RT have been observed by using the cell-sorter protocol [1,2]. Clinical observations are often based on visualizations of tumor lumps using medical imaging. However, for microscopic disease [3,4]; tumors are invisible to modern imaging technologies. Many cell-killing models have been developed and have been extended to microscopic disease [3]. Linear quadratic models based on Poisson statistics have been developed to fit clinical observations [5]. Adjuvant RT [6,7] following surgery has been widely used in many treatment plans. The assumption behind adjuvant RT is that microscopic amounts of tumor tissue may remain after surgery; these must be destroyed to prevent tumor recurrence. Despite extensive studies of tumor controls and the biological effects of RT, little is known about the mechanism of RT than what is thought to be known [8], especially for microscopic disease, due to the limitations of current in-vivo and in-vitro experimental methods.

We found that radio-sensitivity is not a monotonic function over tumor volumes especially for microscopic disease and we show that Poisson statistics-based models can fit clinical data despite that they are wrongly based on the biological behaviors of bacterial cells. We showed that large fluctuations on radio-sensitivity over tumor volumes may not matter clinically, thus validates any Poisson models using cell killing effects over tumor volumes. This also justifies the equivalent radio-sensitivity model on RT. We consider that a tumor cell is a mammalian cell that is not a self-independent complete life organism but a bacterial cell is. A normal mammalian cell differentiates, has limited proliferation, has spatial arrangement, maintaining a healthy cell communication, and would not be recognized by mammalian immune system as alien, while a tumor cell may not differentiates, may proliferates indefinitely, does not have regular spatial arrangement, has malfunctioning cell communication, and may be recognized by the immune system (especially for virus infected cancers). Therefore, we should investigate the effect of RT on tumor cells, with considerations of cellular communications and signaling transductions.

## Methods

### Poisson statistics and cell killing

RT models based on Poisson statistics are supported by clinical data and have become widely accepted for the past half centaury. According to the Poisson model [5,9,10], the probability that a cell receives *m *lethal events is:

(1) *P*(*Y*, *m*) = *Y*^*m*^*e*^-*Y*^/*m!*

where *Y *is the rate of lethal events.

In RT, a cell survives on radiation only if it receives zero lethal events, corresponding to *m *= 0 in (1). The probability that a cell survives on radiation is therefore:

*P*(*Y*, *m *= 0) = *e*^-*Y*^

For a linear model, *Y *is proportional to the dose *D*, with coefficient *α*. For a linear quadratic model, *Y *is not only proportion to the dose but also depends on a quadratic function of the dose D with an additional coefficient *β*. In addition, cell growth occurs between RT treatments; it is assumed that cell proliferation is proportional to the time t between treatments, with another additional coefficient *λ*. Then the rate of lethal events is:

(2) *Y *= *α D *+ *β Dd *- *λ t*

where *D *is the total dose and *d *is the dose per RT treatment, so that *D *= *d f*, where *f *is the number of treatments. The survival fraction of the cells is:

(3) *S *= *e*^-*Y*^

Assuming there are *N *tumor cells before RT, the Tumor Control Probability (TCP) is given as:

(4) $$\begin{array}{c} \text{TCP}={\left({\text{e}}^{-\text{s}}\right)}^{N}={\text{e}}^{-\text{sN}}={\text{e}}^{-{\text{Ne}}^{\left(-Y\right)}}\\ ={\text{e}}^{-{\text{Ne}}^{\left(-\alpha \text{ D}-\beta Dd+\lambda \text{ d t}\right)}} \end{array}$$

It is well known that dose in-homogeneity [3,8-10] has always been an issue in RT models; it turns out that if *D*(*x*) is actually a distribution function of space, then both RT and medical image models become mathematically intractable in many situations. Owning to the remarkable discovery of equivalent uniform dose (EUD) model [9], much of the intractable situations have been solved. For any RT or diagnostic radiation, there exists an equivalent uniform dose that achieves the same clinical result even though the actual dose is not uniform. Niemierko's EUD [9] has been proved correct and effective in RT and diagnostic image models. Therefore, we can use an equivalent uniform distributed dose per fraction in RT regardless the nonuniform dose. In this way the total dose is approximately a discrete function linearly proportional to the time after the start of RT [10]. The cell repopulation constant can be scaled [10] by the number of days or hours per fraction, then

(5) *λ*' = *λ *d t/f

Therefore, the Tumor Control Probability (TCP) is given as:

(6) $$\begin{array}{c} TCP=e^{-Ne^{\text{(-}\alpha \;D-\beta Dd+\lambda \text{ d t})}}={\text{e}}^{-{\text{Ne}}^{\text{(-(}\alpha \text{ D}-\beta Dd+\lambda \;dt))}}\\ =e^{-{\text{Ne}}^{\left(-(\alpha^{\prime }\text{ D})\right)}} \end{array}$$

where

(7) *α*' = *α *+ *β *d - *λ*'

### The Poisson statistics – widely validated models were actually based on bacterial cells

We investigate the biological behaviors of bacterial cells and found that the widely validated Poisson-statistics based RT models were actually based on the bacterial cells rather than human cancerous cells, because those RT models all assumed no cellular communications.

It has been studied for many years that bacterial population is an exponential growth upon available simple energy source (i.e. light, carbon etc). This because bacterial cells do not communication each other and each cell is an independent complete living organism. Models for bacterial cell growth have been developed and we find it coincides with the RT models based on Poisson statistics.

Bacterial cell repopulation is exponentially growth subject to the following differential equation:

(8) dN/dt = *λ *N

In a RT model, the time required to double the amount of cells is called clinical doubling time (CDT) [6] and is an analogue of doubling the bacterial population.

Let N = 2N_o_, from equation (8), then

(9) CDT = log2/e^*λ*t^

(10) *λ *= log2/CDT

where log is the natural logarithm. In RT, CDT is usually a clinically determined parameter. If *λ *is negative in equation (8), then *λ *is denoted as *α*, and is called radio-sensitivity in RT, therefore equation (8) become:

(11) dN/dt = -*α *d

This equation indicates a log kill of cells on RT, which coincides with Poisson statistics. For linear quadratic model, cell killing is a function of the linear and quadratic forms of dose and cell proliferation, therefore,

*Y *= *α D *+ *β Dd *- *λ t*

The above equation coincides with equation (2) in RT models based on Poisson statistics. Cell growth occurs between each fractions of RT, so this effect must be counteracted in the cell killing model. Therefore, *Y *is proportional to dose and quadratic function of dose plus a counter action from cell proliferation. TCP is then given as:

$$\text{TCP}={\text{e}}^{-{\text{Ne}}^{\text{(-}\alpha \text{ D}-\beta Dd+\lambda \text{ d t})}}$$

This coincides with (4) in Poisson statistics.

The cell repopulation constant can be scaled by *λ*' = *λ *d t/df. Then the "TCP" of equation for cell growth become:

$$\text{TCP}={\text{e}}^{-{\text{Ne}}^{\left(-(\alpha^{\prime }\text{ D})\right)}}$$

where

*α*' = *α *+ *β *d - *λ*'

This actually coincides with equation (6) in Poisson statistics.

One may argue that we do not know if there is any intercellular communication among bacterial cells. To answer this, we need to look at metabolism and signal transduction of a bacterial cell. Bacterial reproduction and metabolism depend on simple energy source such as lights; carbons rather advanced bio-organic compounds such as sugar and protein supplies. A bacterial cell genome encodes only a few thousands or even less protein-coding genes (i.e. *Haemophilus influenzae *~1740 genes and *Escherichia coli *~4400 genes) and there is no splicing or alternative splicing in bacterial genome. Bacteria are prokaryotes and even more advanced eukaryotes such as budding yeast (*Saccharomyces cerevisiae*) containing only 6357 genes (among which ~5800 are functional) without alternative splicing. But genes in a tumor cell are coded by human genome and are abundant with splicing and alternative splicing, even though some genes may have been mutated (virus induced cancer cells may contain virus DNA sequences). None of bacterial genes code for complex human transmembrane proteins with a least portions of glycoprotein outside tumor cellular membrane for cellular signaling and immune recognition. In fact, most bacterial cells have hard shells outside their cellular membranes as protections, making that bacterial cells unlikely communicate each others. Furthermore, a bacterial cell does not have the complex signal transduction like a tumor cell rather just simple expression of a few genes such as HST (heat shock protein) upon changing environments such as radiation exposure or heat. A bacterial signal transduction does not have intercellular communications and does not signaling immune recognitions, which are the situations of tumor cells. But the survival of mammalian cells including tumor cells depend on intercellular communications, and sufficient nutrients such as blood circulations, sugar, and protein supplies as well as the expressions and regulations of a number of human genes in a number of pathways, such as apoptosis related pathways. Unfortunately, the widely validated Poisson-statistics based models do not consider the actual biological behaviors of tumor cells. Therefore, we conclude that Poisson-statistics based models are actually based on the biological behaviors of bacterial cells rather than human cancerous cells. The biological mechanisms of Poisson-statistics based RT models are wrong, regardless that those RT models have been validated clinically.

### Calculation of tumour cell distribution upon RT

Since Poisson-statistics based RT models have been widely proved correct clinically, we used breast cancer cumulative Ipsilateral Breast Tumor Recurrence (IBTR) data in [6], which is from five randomized control trials of lumpectomy, with and without radio therapy in the early-stage breast cancer in our investigations. CDT is a standard clinical gauge to estimate the tumor development. For breast cancer, the reported average CDT with small variation is determined as 100 days [6], which is CDT = 100/365 in the unit of year. Therefore, using equation (10), *λ *is determined as 2.3. At any time t, the new total number of cells grown from the original cell number N_o _is therefore given by:

(12) N = N_o_*e^2.3t^

After surgery, if we know the remaining tumor cells, we can estimate how long it will take to grow into a clinically detectable tumor. Although distributions of the remaining numbers of tumor cells right after surgical removal of tumors are not known, they can be determined statistically from the year of tumor recurrence data as following: We use the assumption that in general, observations of tumor recurrence are referred as tumors sized around 1 centimeter in diameter, which corresponds to roughly 10^9 ^tumor cells [6,8]. Let us set N_c _= 10^9 ^as a critical detectable number of cells for a tumor detection. From (12) we get:

(13) *N*_*o *_= *N*_*c*_*e*^-2.3*Tc*^

where *Tc *is time for tumor recurrence to be observed after surgery (i.e. *Tc *= 8 year). Equation (13) determines the distribution of remaining tumor tissues right after surgery from the available follow up tumor recurrence data reported in [6].

## Results

### Fluctuations on radio sensitivities

**Figure 1:**
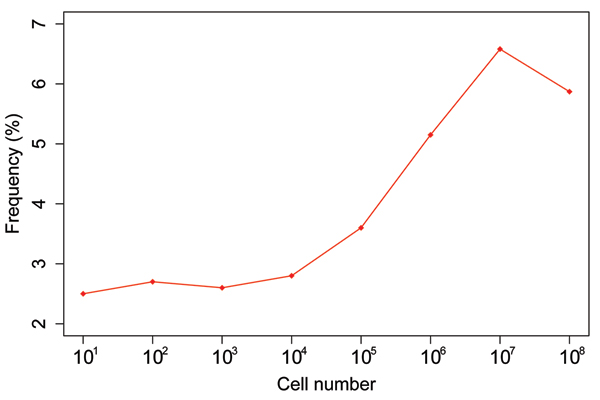
The combined Poisson distribution of remaining cells after surgery. The x-axis represents cell numbers and y axis is the relative frequency.

**Figure 2:**
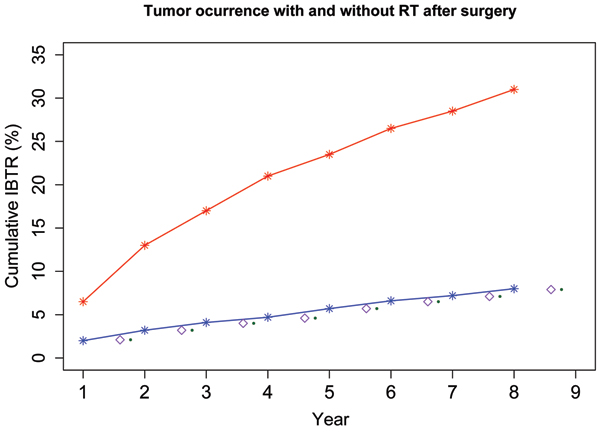
Red line represents tumor re-occurrence rate without RT after surgery while blue line is the rate with RT after surgery. IBTR (Ipsilateral Breast Tumor Recurrence) rate is shown in as purple diamond when a middle point value of the largest and the smaller cell killing rate was used; the rate is shown as green circle when largest cell killing rate was used.

Two sets of results based on the calculation using equation (13) have been obtained, one set refers RT after lumpectomy and the other one is lumpectomy only. We found the distribution of remaining tumor cells after surgical removal of tumors without RT actually coincides with an analytical form given below:

(14) *y*(*x*) = a*(*poisspdf*(*x*, 2.2) + 3/4*poisspdf(*x*, 8))

where a = 23.659 and poisspdf (a standard Matlab ^® ^function in statistics) is the probability mass (density) function of the Poisson distribution. The visualization of this analytical form is shown in figure 1:

Equation (14) is validated by obtaining tumor recurrence rates derived from equation (12). We then plot tumor recurrence rates which are denoted as the red plot in the figure 2. The curve represents the tumor recurrence rates as a function of time (number of years after surgical removal of tumor). From the plot, we can see that at the year 8, the cumulative IBTR is 31%, which fits the data reported in [6].

**Figure 3:**
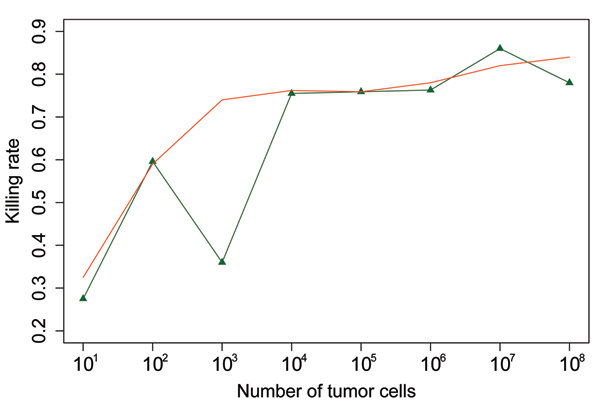
Cell killing effects according to the tumor sizes. The green line represent original cell killing rates over the tumor volumes, while the red line is killing rates over the tumor volumns after smoothing by a combination of Poisson distributions.

From tumor recurrence data, equation (13) gives the both distributions of remaining of tumor cells after lumpectomy with and without RT. By comparing two distributions, cell killing rates of RT on the remaining tumor tissues after surgery are determined as the green line in figure 3.

In the figure 2, we also plot IBTR with radio therapy as shown in the blue line. At year 8, IBTR is reduced from 31% without RT to 8% with RT, which matches exactly the clinical observed statistics that was reported in [6].

This analysis shows that under the combined form of Poisson distributions in (14), roughly 2/3 of patients do not have a remaining tumor cell after the surgery and presumably no recurrence of tumor. Only about 1/3 of the patients may have remaining microscopic tumors after lumpectomy of breast cancer.

As visualized in Figure 3, it is observed that tumor tissues of size 10^7 ^cells have largest cell killing rate on RT while 10 Remaining tumor cells after surgery gives the smallest cell killing rate. It appears overall cell-killing rates varies significantly according to tumor sizes for the microscopic diseases but in general it shows a tendency that, the larger the tumor size, the higher killing rate is. This is obviously contradictory to current published literature and the commonly accepted conventions. Current RT models have valid explanations on RT with a conclusion that the larger the tumor is, the lesser cell killing rate is, due to larger hypoxia effects on larger tumor upon RT. It might appear that we have violated above proved conclusion on RT. We would also actually correct because the conclusion should not extend to microscopic diseases. An enormous issue here is that would the large fluctuation on cell killing rates affects the clinical outcome and would not matter somehow? Otherwise either we or the RT models based on Poisson-statistics are wrong! In the next sub-session we show that our contradictory conclusion and the Poisson-statistics based RT models can both fit clinically data well.

### Clinically correct – biologically wrong models

We have shown that RT models using Poisson-statistics are actually based on bacterial cells without considering tumor cellular signaling. In this sub-session, we show that the fluctuation on radio-sensitivity although significantly varies may not matter too much in clinical outcomes. Fluctuation can be smoothed by a combination of Poisson distributions as:

(15) *Kill *= 3*(*poisspdf*(*x*, 3.5) + 2*poisspdf(*x*, 9))

Equation (15) is illustrated by the red curve in figure 3. The green line represent original cell killing rates over the tumor volumes. It shows radio-sensitivity is in general an increasing function over tumor volumes for microscopic diseases.

To test the validation of (15), we use equations (9) and (12) to calculate the IBTR. We find it matches clinical data very well and is also plotted as the blue line in figure 2. It shows at year 8, the tumor recurrence rate is also accumulated to 8% as reported in [6]. Therefore, the equation of cell killing rate in (15), although coming with much less fluctuation, is an increasing function of tumor volumes, and does not affect much on the clinical outcomes.

In Poisson-statistics based RT models, constant radio sensitivities are sometime used. We also investigate constant radio sensitivities to validate the Poisson-statistics based RT models. We can use a middle point value of the largest and the smaller cell killing rate. The IBTR is then plotted as the purple diamonds in figure 2. If we used the largest cell killing rate, IBTR is plotted as green circle in figure 2. Both situations give 8% of IBTR at the year 8. It is now can be concluded that the large fluctuation of radio sensitivity does not give obvious clinical difference. Using constant radio-sensitivity in a tolerate range gives roughly same clinical outcome. Therefore, a Poisson-statistics based model using a constant radio-sensitivity can be correct clinically.

Comparing the two groups (with RT and without RT) of IBTR data only gives us the total cell killing rates on the completed RT. In real RT, dose is given by fractions. We want to estimate the cell killing rates per fraction. Let us assume the total dose is divided into just 10 fractions (usually in real RT plan, there are more than 10 or even 20 fractions). From figure 3, we can see the largest cell killing rate per fraction is less than 17% (if we use 20 fractions, the cell killing rate per fraction is much smaller than 8%). This largest killing rate corresponds to the microscopic tumor tissue size of 10^7 ^tumor cells. This largest 17% is significantly smaller than other reported in literatures such as a popular 40% of cell killing rate per fraction. This raises another contradiction.

**Figure 4:**
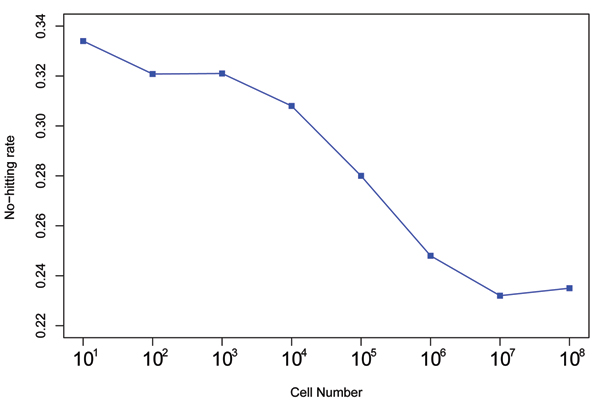
**Another sample figure title**. Ratios of effective non-hitting cell distributions, x-axis is tumor size and y axis is probability.

A number of Poisson-statistics based RT models use the concept of non-hitting cells upon radiation. If this is true; and if we use 40% constant cell killing rate per fraction, then the percentages of non-hitting cells are ranged between 23% and 34% as plotted in figure 4. It appears that tumor size of 10 cells receives largest hitting rate while the non-hitting rate varies significantly over tumor sizes. Such large variations violate the assumptions of Poisson-statistics based RT models, which non-hitting rate should be a constant over tumor volume. There is no statistical reason why non-hitting rates should be fluctuated so significantly over tumor volumes. We further investigate the cell killing effects in the next section.

Since we use two set of IBTR data in [6], one is the tumor recurrence with adjuvant RT, and the other one is no RT, then we determine cell killing rates from cell distributions by comparing the calculated results from RT and without RT after surgery. We do not have to consider the quadratic form or tumor proliferation explicitly as they have already been included in equations (6) and (7). We have shown that although cell killing rates vary significantly, Poisson-statistics based RT models using constant radio sensitivity can be correct. Now we can conclude that we can use an equivalent uniform radio-sensitivity model that can be clinically correct. The coefficient *α*' in equations (6) and (7) can be considered as an equivalent radio-sensitivity, because it can be used to achieve same clinical results compared to more sophisticated linear – quadratic – proliferation models, as far as there exists a dose that can be considered uniform per fraction in RT. Even though uniform dose distribution can not be achieved clinically, we still can use the simplified model as an equivalent to more complicated models in the situations with quadratic form and cell proliferation included. Because in real RT, dose can never be absolutely uniform, however, Niemierko's Equivalent Uniform Dose (EUD) [9] has solved the main issues in RT. Non-uniform dose somehow may not affect the radio sensitivity. We have shown a case that fluctuation of radio-sensitivity does not matter in fitting clinical data. As far as somehow equivalent uniformly distributed dose is valid, equations (6) and (7) are valid. We name this variant of RT model as an equivalent uniform radio sensitivity model, as it utilizes a cornerstone in RT – the Niemierko's EUD and other techniques [9,10]. The successes of Niemierko's EUD in fitting clinical data also support the validation of the above equivalent uniform radio sensitivity model.

## Discussion

### Clinically correct – biologically wrong models

We find that radio-sensitivity is proportional to tumor size on microscopic diseases, which is evidently contradictory to the common conclusions in RT models. We show that a large fluctuation on radio-sensitivity may not matter and may give the same clinical outcome. We validate the clinical correctness of RT models based on Poisson-statistics using constant cell killing rates, regardless their wrong biological mechanism that has been based on the bacterial cells. This also validates the equivalent radio-sensitivity model. Utilizing the concept of non-hitting cells, many RT models have fitted the clinical data well. In physics, de Broglie's wave-particle duality indicates any radiation is also subject to Heisenberg's principle of uncertain. There are also statistical proofs that some cells may not be hit by radiation during RT. However, we consider a cancer cell is so huge compare to bacteria cell, a question here is if no-hitting cells would ever exist in RT? To answer this, we need to know the normal exposure of radiation among humans. The total life time exposure of radiation of all sources is less than 0.1 Gy in average and the virtually all cancers in atomic bomb survivors received more than 0.1 Gy with a majority received more 1 Gy. It appears 0.1 Gy dose of radiation can induce cancer, and actually Michael Joiner et. al. [1,2] have verified the hyper sensitivity of RT at low dose (i.e. < 0.2 Gy). This means a low dose such as 0.1 Gy give relatively higher radio-sensitivity on cell killing and is considered as a factor to induce cancer. If a RT treatment is divided into fractionated RT with a low dose per fraction, each fraction is typically 2 Gy or more. Let us consider a very conservative estimation of RT, in a case just one dose of 1 Gy, then the biological effects on this just one time 1 Gy dose are: there are more than 20 thousands ionization per cell, more than 100 DNA stands breaks per cell with obvious chromosome changes and differential gene expressions and regulations. If this 1 Gy is a high linear energy transfer radiation, 1% of cells survive, with 30 transformations per million cells, while if low linear energy transfer, 90% percentage of cells survive with 3 transformations per million cells. Therefore, it is evident that 1 Gy dose offers severe damages on each cell, but how many cells are killed depend on whether low or high linear energy transfer. It is obviously not possible that there exists even one single non-hitting cell on RT. Such non-hitting cells may possibly happen on lower radiation dose such as 0.1 Gy or less, but definitely not at a dose of RT. Even though the percentage of killed cells varies from 1% to 90%, all cells are affected significantly upon 1 Gy 0.1 Gy, humans must not have a mechanism to tolerate radiation of 1 Gy dose, otherwise theory of evolution can not be valid. Upon RT, even a smallest dose such as 1 Gy RT affects all cells and counts to the risk of radiation induced secondary cancer. Cells that are not killed in RT do not mean they are not hit, but they are all damaged or at least affected somehow and may later proliferate indefinitely with malignant transformation in the offspring. Because telomerase can be damaged by radiation, at the reactivation of telomerase, cells may become immortal just like cancer cells and may undergo malignant proliferation. We have measured levels of telomerase and found telomerase is related to the malignant transformation [4,11]. Not non-hitting cells and not killed cells should be two different concepts in RT. Upon RT, all cells are all hit, some are damaged and some are killed. Damaged cells are possibly related to tumor recurrence, genomic instability, bystander effects, tumor malignant transformation and metastases. Those survived tumor cells can signal to the surrounding both normal and tumor cells to develop blood vessels and to help re-generate killed tumor cells. Non-hitting cell models in RT are just models based on phenomena; they should not be interpreted as there are real non-hitting cells in RT. Joiner et. al. [1,2] reported that radio-sensitivity is not a monotonic function on small doses – a phenomenon called low-dose hyper radio-sensitivity. This has inspired us to find that radio-sensitivity is also not monotonic function of tumor volumes especially for microscopic diseases.

As for the genomic instability, it originally refers to induced long lasting sub-lethal effect or normal cells turning into cancer while our view of genomic instability is the change of gene regulation that cause abnormal differentiation of original cells, that maybe related to malignant transformation or induced apoptosis. After RT, cancer cells can be genomic instable as well, not just normal cells. As regard to the original bystander effect, it means non-hitting cells die or mutate as the result of adjacent of hitting cells on RT. Our new view of this effect is different; there exists no possibility on even one single non-hitting cell on RT, bystander effect maybe actually the deconstruction of tumor cellular signaling pathways.

### Cancer cells do have signaling transductions

Traditional RT models do not consider cell communications. We show that Poisson-statistics based RT models were actually based on wrongly bacterial cells of exponential growth or log cell killing on radiation without considering cellular communications. Despite that, Poisson-statistics based RT models fit clinical data well, yet mammalian cells including cancer cells must maintain their signaling transduction pathways or they cannot survive. The large fluctuations of radio-sensitivity are resulted from the disturbances of the signaling pathways although they might not affect the clinical outcome of TCP. We have shown that there is not even one non-hitting cell in RT, then the questions is why some cells are killed, some are not. We consider that it is also related to the cellular signaling and communications. Back many years ago, Dr. Tikvah Alper first proposed that damages on cell membrane on RT can trigger the process of damages on cell membrane on RT can trigger the process of apoptosis, later Dr. Niemierko also gave biological effects of IMRT. Therefore, a tumor cell can be killed by disturbing its signaling transduction or can survive if alternative signaling transductions can be maintained upon RT. It is not only that human genome is abundant with splicing and alternative splicing, but also many proteins are multi-functional [12]. It is possible alternative spliced genes can be expressed and alternative functions of proteins can be carried upon RT. At adjuvant RT, since the remaining tumor tissues are microscopic, thus the underlying distributions of microscopic tumor cells can vary significantly on sizes that affect the tumor cellular communication and signaling transductions.

Tumor cellular signaling transductions are also shown in the human immune system reactions on tumor cells. The effects are obvious on virus-induced tumors, which are recognized by immune system immediately. A tumor cell does communicate with outside with a cellular identification that is depending on the glycocalices located on the tumor cell membrane with at least a portion in the extra-cellular space (outside the cell membrane) for immune cell recognitions. All four types of cell communications have been found in tumors: endocrine signals, autocrine signals, Paracrine signals and Juxtacrine signals. All four types of tumor cellular communications support Niemierko's theory on repairing damaged cells by un-killed neighboring cells upon radiation [13]. Tumor cell-to-cell communications are often made by direct contact usually via the interaction of the glycocalices of the tumor cellular membrane. Tumor cells are mammalian cells which are affected by the intra-inter- and extra-cellular signaling to receptors that face outwards from the membrane and trigger the gene regulation inside cells. Therefore disturbing the tumor cellular communications by RT can kill or affect the survived tumor cells. GJIC (gap junction intercellular communications) are abnormal in tumors and are not functioning, yet there are still some signals through the transmembrane (TM) and the ion-channels in tumor cells.

Although almost all tumors are found to have disrupted or abnormal GJIC, tumor cells are still mammalian cells with cellular communications and regulations, otherwise tumor cells can not survive. Strictly speaking, Poisson-statistics based models are incorrect biologically and that is why Niemierko's EUD and his sequential development of clustering algorithms [13] are so important in developing correct RT plans. We present an equivalent uniform radio-sensitivity model based on Niemierko's EUD. We find that TM and intrinsically unstructured proteins (IUP) are correlated and co-exist in cell membranes, and tend to have opposite physiochemical prosperities [14,15]. Niemierko's theory also shows that survived cell cluster can repair the damage cells with a radius depending on the size of the cluster.

In RT models, cancer cells are considered as immortal and RT is aimed to achieve TCP = 100% so that a treatment plan must achieve to kill all immortal cancer cells, otherwise cancer will come back, even one tumor cell was not killed. As matter of fact, cancer cells are not immortal at all and all cancer cells must die without exception. They just proliferate indefinitely and faster than normal cells so it better to describe cancer cells are uncontrolled fast-growing cells. Normal cells maintain cross-linked signaling pathway networks, which synergize health control of cell metabolism and growth, while for cancerous cells, one of the signaling pathways is damaged and thus result loss of control of a signaling pathway in the networks. Cancer cell survival is also promoted by blocking apoptosis via the ras/phosphoinositol/Akt pathway, and such pathway can be affected by RT. It is often unknown how radiation alters regulatory pathways, yet it has been shown that several types of cancer are related to co-regulations of bidirectional promoters [16]. Actually it is less meaningful to estimate how many percentage of cancer cells are killed by RT rather it is more meaningful how malignancy and proliferation get altered or controlled by RT. Because any cancerous cells die anyhow, the control on malignancy and proliferation determine prognoses of patients upon RT.

Tumor cell-to-cell communications can be made through although abnormal gap junctions, which allow different molecules and ions to pass freely among tumor cells throughout TM channels to keep tumor tissue well nutritive Disturbing the TM signaling process can result the death of tumor upon RT. Larger tumor tissue with a higher spatial cell density would experience higher chances of blocking the cell-to-cell communication upon RT because some adjacent tumor cells had been killed, thus cell-to-cell communication had been destroyed more severely. Research has shown that closing gap junctions, involved in GJIC had led to an increased cell killing by the bystander effect. Furthermore it has been reported that extra-cellular signaling pathways had been identified as an integrator of multi-cellular damage responses preventing tumor development through the removal of damaged cells and inhibition of neoplastic transformation. Larger tumor cluster with higher density statistically experiences a larger portion of destruction of multi-cellular communications among tumor cells thus effectively suffers larger cell killing rate upon radiation.

Recent research showed that under appropriate micro-environments, human breast cancer stem cells could be induced to express their connexins and start to partially differentiate. This gives a clue that early tumor cells may still have telomerase activity and grow inhibition. Tumor cells are physically contacted via their membranes, upon RT, a larger cluster of tumor cells are killed more significantly than complete isolated tumor cells because of more server damages on tumor cellular communications.

Radiation can induce genetic or epigenetic change in a single-hit cell altering the internal homeostatic control of transcriptional regulation of the genome, thus may generating additional hits. Although this study has been conducted for radiation-induced cancer [3], non-targeted and delayed effects of exposure to ionizing radiation contribute to the death of single hit tumor cells that influence the survival of surrounding tumor cells. There is no doubt about the how genomic instability occurs upon radiation, evidences have also been found in fibroblast systems of the involvement of GJIC in bystander responses. Based on our different definitions of bystander effect and genomic instability given before, those evidences support our statement that smaller tumor tissues receive less cell killing rates than larger tumor tissues because of the difference of underlying probability density distributions of tumor cells in tumor tissues for microscopic diseases. Furthermore, tumor cells emit signals to the surrounding normal tissue to generate blood vessels to help tumor development, survival of a tumor tissue also depends on its sufficient blood vessels and blood circulations. Destroying tumor blood vessels may result the death of a whole tumor tissue. This indicates that larger tumor tissues cluster should have larger cell killing rates on RT, because blood vessels are also likely destroyed upon RT.

Finally Radio-Immuno-Therapy (RIT) built on the cytotoxic potential of monoclonal antibodies through the addition of a radiation has been used successful to deliver a therapeutic dose of radiation, involving the combination of a monoclonal antibody directed against a specific antigen with a source of radiation. The mechanism is that cancer cells can trigger human immune reaction while normal cells do not. Therefore, larger cancer tissue should trigger stronger human immune reaction. For RT, it means a larger cell-killing rate than that of a smaller tumor tissue. For a large microscopic tumor tissue, RT may more likely trigger the apoptosis related pathways by more severely disturbing tumor cellular communications, and RT works more effectively with human immune system to achieve larger killing effects, while for a completely isolated tumor cell, synergy of those effects would not occur.

## Conclusion

Clinical speaking, our limitation of current technology has not enabled us to determine any amount of remaining tumor cells after surgery [3]. Surgeons always hope that there is not even one tumor cell remaining after removing any visible tumor tissue. Scientifically, it is possible that there are still invisible remaining microscopic tumor tissues, but we do not have a way to verify that. The remaining tumor cells can range from 1 to 10^8 ^and are not clinically detectable. They are referred as microscopic diseases by Suit and Niemierko's laboratory that reported an offset of 12 Gy from macroscopic tumors in TCP [3]. The possible remaining tumor cells after surgery accounts for tumor recurrence. Adjuvant RT is often performed after surgery and it is hard to verify the real process of cell killing in microscopic diseases. Different RT models have been developed and many parameters had been justified for model fittings. It has been commonly regarded that upon RT, the larger the tumor tissue is; the less cell-killing rate is. We find that for microscopic diseases, actually, larger tumor tissue receives larger cell killing and radio-sensitivity is not a monotonic function. We use the available IBTR data to calculate the cell killing rates upon RT and show that common conclusions on cell killing can not be extended to microscopic diseases. However, we also demonstrate that the large fluctuations on radio-sensitivity may not matter and Poisson-statistics based models can be correct clinically regardless of their wrongly biological mechanism based on bacterial cells. RT and human immune system can work together to kill tumor. cells. We oppose the concept of non-hitting cells in RT models. Our results are based an equivalent uniform radio-sensitivity model that utilizing the concept of Niemierko's EUD, we found that smaller tumor tissue may experience less killing rate in adjuvant RT which is also based on Niemierko's theory that un-killed surviving cell clusters can repair/regenerate killed cells within a radius R from each of cluster center [13]. It is commonly regarded that tumors can be resulted from the DNA double strand breaks and mutations; we believe that tumors are resulted from abnormal expressions and regulations, while mutations count for that. It has been widely verified that RT break the double DNA chains and tumor cells are killed because they can not repair the damages on the DNA chains upon RT. We believe that the energy to break DNA double strands for the above killing upon RT is high, tumor cells can be killed by disturbing their intercellular communications and the energy for that would be much lower than breaking the DNA chains with irreparable strength.

Tumor characteristic specific anti-genes no matter found or not may exist and if so, may roughly proportional to the size of tumor tissue. For some cancers, they can be detected by patients' blood and urine tests. Based on the clinical outcomes, if an existing tumor tissue is too tiny, blood tests may not detect any tumor specific antigenes, indicating that patient's immune system may not be stimulated and the patient's body has not recognized the invasion of a tumor yet, therefore tumor specific anti-bodies may not have been produced yet. As tumor tissue grows bigger, the immune system may begin to recognize the invasion of tumor and thus may produces anti-bodies as a result of tumor. For microscopic disease, if the remaining tumor tissue after surgery is considerably large (still invisible), patient's immune system works more effectively with RT to give a larger cell killing rate than "clinical infinitesimal" tumor tissue that the RT may work alone without the synergy of immune system.

The aggressive biological behaviors, tumor cellular communications and biological mechanism of any seed cancer cell or a small cluster of beginning cancerous cells would support the finding that smaller tissues receive less cell-killing rate on microscopic diseases. Although this obviously contradict to some proved conclusions on RT that large tumors are more resistant to radiotherapy due to the higher level of hypoxia, it does mean the proved conclusions can not apply to tumors of microscopic diseases (invisible tumors), because tumor cells behave differently than simple bacterial cells. Cancer cells do communicate each other. A killed tumor cell upon RT can trigger the death of neighboring tumor cells and therefore, statistically, a larger tumor tissue receives larger cell-killing rate than a tiny isolated tumor tissue, because RT significantly destroys large-cluster of cellular communications that may affect genomic stability and may trigger the apoptosis of tumor cells. This also explains that a completely isolated tumor cell may experience less cell killing effect on adjuvant RT than larger tumor tissues for microscopic diseases. However as tumor tissue become larger and larger and eventually visible, human immunize system may begin to take significant roles, then the hypoxia effect dominate the cell killing rate on RT. An inverse conclusion can be reached for tumors of microscopic diseases. It can be concluded that radio-sensitivity is not a monotonic function on tumor size upon RT; it is proportional to the size of tumor tissue on adjuvant RT while it decreases on larger visible tumors.

## Competing interests

The authors declare that they have no competing interests.

## Authors' contributions

Jack Yang and Andrezej Niemierko conceived project and designed the experiment. Jack Yang and Mary Yang performed the experiment. Youping Deng helped manuscript writing and analysis. Andrezej Niemierko guided the project.
